# Exercise as Medicine: Quantifying the Effects of Physical Activity on Fibromyalgia Pain—A Systematic Review and Meta-Analysis

**DOI:** 10.3390/brainsci16040365

**Published:** 2026-03-28

**Authors:** Vasileios T. Stavrou, Panagiotis Zis

**Affiliations:** 1 Medical School, University of Cyprus, 1678 Nicosia, Cyprus; vasileiosstavrou@hotmail.com; 2 Unique Safe Tele-Exercise Project—Institute for Digital Health and Chronic Diseases, 41336 Larissa, Greece

**Keywords:** pain reduction, exercise therapy, physical activity

## Abstract

**Highlights:**

**What are the main findings?**

- Exercise interventions reduce pain in fibromyalgia patients across multiple modalities.
- Cumulative MET-based workload shows no consistent monotonic dose–response pattern.

**What are the implications of the main findings?**

- Exercise prescription should prioritize tolerability, pacing and program structure over dose escalation.
- A symptom-responsive, Talk Test–guided web calculator can support the planning of individualized weekly exercise programs, including people in remote or underserved settings.

**Abstract:**

Background: The pain experienced by people with fibromyalgia (FM) is thought to be the result of altered nociceptive processing, impaired descending inhibition and reduced tolerance to physical load. However, the relationship between the amount of exercise and pain reduction remains unclear. Methods: This study synthesized randomized controlled trials of exercise interventions for FM to quantify the combined analgesic effects of different types of exercise. A secondary aim was to standardize exposure using metabolic equivalent of task (MET)-based metrics and examine the association between cumulative intervention dose (MET·h) and analgesic response (Hedges’ g) across intervention arms. Following the PRISMA guidelines, a search was conducted in PubMed for randomized controlled trials published up to 31 December 2025. After screening and a full-text assessment, 15 trials were included. The protocols were converted into MET-defined intensity and weekly MET·min exposure, and the cumulative dose was calculated as the total MET·h accrued over the intervention period. Random-effects models were used to estimate the pooled effects within modality subgroups. Results: Across modalities, exercise was associated with reductions in pain, with effects typically falling within the small-to-moderate range. Larger improvements were observed in structured or supervised programs. The dose-response scatter plot showed wide variability across the dose range, with overlapping confidence intervals. An exploratory fourth-degree polynomial fit explained limited variance (R^2^ = 0.1615) and did not indicate a monotonic dose-response pattern. This suggests that cumulative workload alone is a weak proxy for therapeutic response. Conclusions: Based on these findings, a pain-responsive algorithm combining weekly Visual Analogue Scale (VAS), ΔVAS and Talk Test thresholds was implemented as a preliminary online calculator to support the prescription of exercise tailored to symptoms.

## 1. Introduction

Fibromyalgia (FM) is a chronic multi-symptom pain syndrome characterized by widespread musculoskeletal pain. It is commonly accompanied by fatigue, non-restorative sleep, cognitive impairment and mood disturbances. It is currently conceptualized within a biopsychosocial framework as a disorder involving altered central pain processing and modulation [1]. As a non-nociceptive pain condition, FM is associated with central sensitization, which manifests as increased excitability in spinal and supraspinal pathways, and reduced efficacy of descending inhibitory control. These mechanisms contribute to clinical hyperalgesia and allodynia, as well as symptom flare-ups that are disproportionate to peripheral nociceptive input [2,3]. The central sensitization model of FM posits amplified nociceptive processing alongside reduced efficacy of descending inhibitory control [1]. This is embedded within broader autonomic and hypothalamic–pituitary–adrenal (HPA) axis dysregulation, as well as psychosocial influences that shape symptom flares and functional impairment. Psychophysical meta-analytic evidence demonstrates compromised endogenous pain modulation in FM, characterized by attenuated conditioned pain modulation (CPM) and enhanced temporal summation—objective markers consistent with underperforming descending inhibitory pathways [4,5]. Further methodological studies show that CPM impairment is evident across paradigms (e.g., cold pressor versus ischemic pressure) and that poorer CPM is associated with greater clinical pain and disease burden. This strengthens the link between laboratory indices and symptom severity.

Exercise is a core non-pharmacological treatment for FM recommended by international guidelines, with an emphasis on personalized, graded programs targeting pain, function and health-related quality of life [3]. For example, aerobic exercise has the strongest evidence base, with a Cochrane review finding that it probably improves health-related quality of life and may reduce pain and improve physical function when performed at a tolerable intensity and increased gradually [6]. Meanwhile, resistance exercise improves multidimensional function, pain, tenderness and strength over ~16–21 weeks and can be performed safely by most women with FM when the intensity is adjusted and the technique is supervised [7]. Moreover, there is low-to-moderate quality evidence that aquatic and stretching exercises benefit symptoms [6,8]. Mind–body exercise (e.g., Tai Chi) has been shown to have a positive effect on pain [9]. Due to the varied nature of FM syndrome, exercise prescriptions should be tailored to the individual, considering their fitness level, symptom severity, psychological profile and personal preferences. This will maximize adherence and long-term benefits [7,10].

The aim of this systematic review and meta-analysis was to examine the effects of exercise interventions on pain in people with FM. Chronic pain can significantly hinder participation in physical activity and may result in premature termination of exercise. The primary objective was to summarize randomized controlled trials investigating structured exercise interventions in individuals with FM, and to quantify their impact on pain intensity. A secondary objective was to standardize exercise exposure using metabolic equivalents (METs) to explore potential dose–effect patterns between standardized exercise dose (MET-based exposure) and changes in pain. A further aim was to develop a clinically applicable exercise prescription algorithm that responds to pain and is based on weekly Visual Analogue Scale (VAS) scores, ΔVAS and Talk Test thresholds.

## 2. Materials and Methods

This study was a systematic review of randomized controlled trials (RCTs) examining the effects of structured exercise interventions on pain in individuals with FM. This systematic review was reported in accordance with the PRISMA 2020 statement. The review protocol was not prospectively registered (PRISMA 2020 item 24a). The completed PRISMA 2020 checklist is provided as Supplementary Material.

### 2.1. Search Strategy

A structured search of the PubMed database was conducted from its inception until 31 December 2025, using a combination of keywords relating to fibromyalgia, pain assessment and exercise interventions (e.g., aerobic, resistance, aquatic and rehabilitation exercises). The results were restricted to randomized controlled trials within the specified timeframe. The reference lists of the studies included were also screened to identify additional eligible trials. Only peer-reviewed full-text articles were considered.

### 2.2. Eligibility Criteria

Studies were included if they met the following criteria: recruited adults diagnosed with FM according to recognized diagnostic criteria [11], evaluated a structured exercise program, reported sufficient details of the exercise prescription to allow dose estimation (frequency, duration, and intensity), and assessed pain using the VAS, a widely used instrument for quantifying subjective pain intensity on a continuous scale, with mean ± standard deviation (M ± SD) values at baseline and post-intervention. Trials that did not provide adequate information to estimate the metabolic equivalent (MET)-based dose were not considered in the dose–response interpretation.

### 2.3. Study Selection

**Figure 1:**
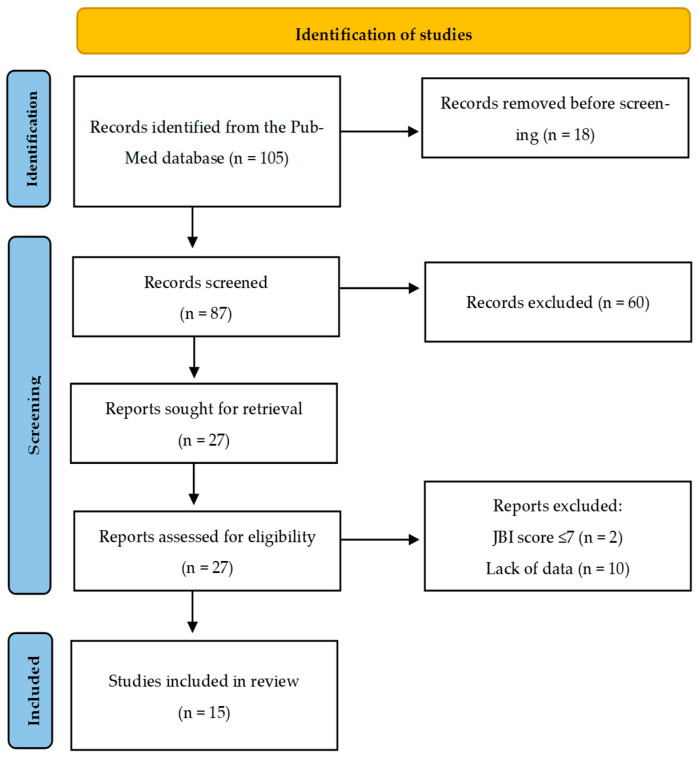
PRISMA flow chart of study selection process. After duplicate removal (*n* = 18), two-stage screening was performed: title/abstract screening (*n* = 87) and exclusion (*n* = 60). This was followed by a full-text assessment (*n* = 27). A total of 15 studies were included in the final review.

Duplicate records were removed prior to screening. Two reviewers independently screened the titles and abstracts of the remaining records, followed by a full-text assessment against the inclusion/exclusion criteria. Any disagreements were resolved through discussion and consensus. The selection process is presented in the PRISMA flow diagram (Figure 1). Overall, 15 studies met the eligibility criteria and were included in the final synthesis.

### 2.4. Data Extraction

Two reviewers extracted data using a standardized template to capture the following information: author, year, country, sample size per group, participant characteristics, exercise modality, intervention duration (in weeks), weekly frequency, session duration, reported intensity, and VAS pain outcomes. Disputes were resolved by consensus. For multi-arm trials, each eligible exercise arm was treated as a separate entry in dose analyses if the outcome data were reported independently.

### 2.5. Exercise Dose Standardization

**Figure 2:**
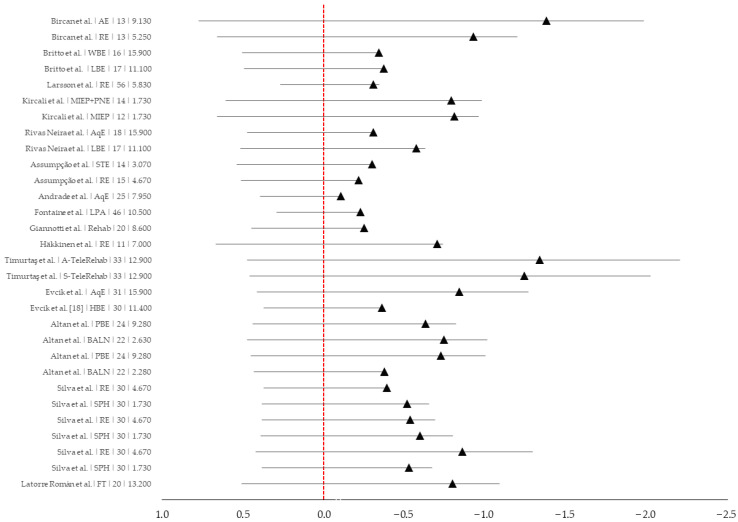
Forest Plot of Analgesic Effect Sizes across Exercise Interventions in Fibromyalgia. Negative values indicate reductions in pain intensity. Triangles represent effect-size estimates for each intervention arm, and horizontal lines represent 95% confidence intervals. The vertical dashed line marks the null value. In the left panel “Study|Protocol|n|MET·h/week” indicates the study/intervention arm, the exercise protocol, the number of participants in that arm, and the weekly exercise dose expressed as MET-hours per week, respectively. Abbreviations: AE = aerobic exercise; AqE = aquatic exercise; A-TeleRehab = asynchronous telerehabilitation; BALN = balneotherapy; FT = functional training; HBE = home-based exercise; LBE = land-based exercise; LPA = lifestyle physical activity; MIEP = motor imagery-based exercise; MIEP+PNE = motor imagery-based exercise protocol and pain neuroscience education; PBE = pool-based exercise; RE = resistance exercise; Rehab = rehabilitation; SPH = sophrology; S-TeleRehab = synchronous telerehabilitation; WBE = water-based exercise.

The Compendium of Physical Activities [12] was used to convert exercise intensity to metabolic equivalents (METs). The weekly exercise dose was calculated as MET·min/week (MET value × minutes per session × sessions per week). The cumulative intervention dose was calculated by multiplying the weekly MET·min by the total number of intervention weeks (cumulative MET·min) and is presented as MET·h in Figure 2 for readability purposes (MET·h = cumulative MET·min/60). Where MET values were not explicitly reported, estimates were derived from the reported modality and intensity category according to Compendium classifications. Studies with insufficient information to derive a reasonable MET estimate were excluded from dose–response analyses. For clarity, absolute intensity was categorized using conventional MET cut-points: sedentary/rest: ≤1.5 METs; light: 1.6–2.9 METs; moderate: 3.0–5.9 METs; vigorous: ≥6.0 METs. Where exercise intensity was not explicitly reported, MET values were estimated based on the exercise modality described and its corresponding intensity classification in the Compendium of Physical Activities. In such cases, the assigned MET values should be considered approximate rather than exact.

### 2.6. Methodological Quality and Risk of Bias

Two reviewers assessed the methodological quality and risk of bias using the Joanna Briggs Institute (JBI) 13-item critical appraisal checklist for randomized controlled trials (Table 1). This covered the following areas: clarity of the research question, suitability of the design, adequacy of randomization and blinding, reproducibility of the intervention, completeness of follow-up, appropriateness of statistical analyses, use of intention-to-treat analysis, reporting transparency, consideration of confounders, disclosure of funding and conflicts of interest Each item was rated as ‘Yes’, ‘No’, or ‘Unclear’. Quality ratings informed the interpretation of the results but were not used to exclude studies. Studies were then classified as: High quality (score: 11–13), Moderate quality (score: 8–10), and Low quality (score: ≤7) [13]. Disputes were resolved by consensus.

### 2.7. Data Synthesis

Due to the heterogeneity of exercise modalities, intervention characteristics, and reporting, the results were synthesized narratively. The synthesis emphasized exercise type, intervention duration, weekly and cumulative MET-based dose, and the magnitude of pre–post change in pain. Dose–response patterns were explored descriptively to assess whether greater pain reductions were associated with higher cumulative MET exposure.

### 2.8. Statistical Analysis

The effect sizes for the pain outcomes were calculated as standardized mean differences based on the change in VAS score from baseline to the follow-up stage within each eligible exercise group (Cohen’s d) and were then converted to Hedges’ g to correct for small-sample bias. Negative values indicate pain reduction. For each effect size, 95% confidence intervals were calculated. Pooled effects within exercise-modality subgroups were estimated using a random-effects meta-analysis approach. Between-study heterogeneity was assessed using Cochran’s Q and the I^2^ statistic, and the corresponding *p*-values were reported. Forest plots were generated for each exercise modality subgroup to display the individual and pooled effect estimates. To explore the relationship between total workload and analgesic response, the cumulative intervention dose was expressed as the total MET·h accrued over the intervention period (MET·h = cumulative MET·min/60) and plotted against Hedges’ g across the intervention arms. An exploratory fourth-degree polynomial trendline was then used to visualize potential non-linear patterns, and these were summarized using R^2^. Meta-analyses were performed using IBM SPSS Statistics 21 (IBM Corp., Chicago, IL, USA).

## 3. Results

### 3.1. Study Selection and Characteristics

The literature search identified 105 records. After removing 18 duplicate records, 87 titles and abstracts were screened, resulting in the exclusion of 60 records. The full texts of 27 reports were assessed for eligibility and 12 were excluded (JBI score ≤ 7, n = 2; insufficient data, n = 10). This resulted in 15 randomized controlled trials being included in the final synthesis (Figure 1). The included trials were published between 2001 and 2025, involved adults with FM and had sample sizes ranging from 11 to 56 participants per exercise arm. Interventions across studies included aquatic/pool-based exercise, resistance training, functional or lifestyle-based physical activity programs, rehabilitation/telerehabilitation formats and multimodal or cognitive-based exercise protocols. The intervention delivery formats varied across the studies, with the majority comprising in-person, supervised programs. A smaller number of trials adopted home-based and telerehabilitation approaches. This variability should be considered when considering the applicability of the findings to different clinical and digital care settings.

### 3.2. Analgesic Responses Among Exercise Modalities in FM

In addition to the descriptive synthesis at the level of each intervention arm, pooled effects were estimated for each exercise-modality subgroup using random-effects meta-analysis. A forest plot summarizing the intervention-arm effect sizes is presented in Figure 2, and between-study heterogeneity is reported using Cochran’s Q, the I^2^ statistic, and the corresponding *p*-values.

#### 3.2.1. Aquatic Exercise

Across the six aquatic and pool-based intervention arms (Table 2; AqE, PBE, WBE), exercise intensity was categorized as moderate according to the Compendium-assigned MET value for aquatic exercise (5.3 METs). Protocol duration ranged from 4 to 24 weeks, with weekly exercise volume ranging from 477 to 954 MET·min/week (7.95 to 15.90 MET·h/week) and cumulative dose ranging from 3816 to 13,356 MET·min (63.6 to 222.6 MET·h). All interventions were associated with reductions in VAS pain, although the magnitude of improvement varied across studies. Standardized within-arm pre–post effects ranged from small (Cohen’s d = −0.11) to moderate-to-large (up to d = −0.86), while absolute pain reduction (ΔVAS, post–baseline; negative values indicating improvement) ranged from −0.40 to −2.52. The pooled random-effects estimate for the aquatic subgroup indicated a small-to-moderate analgesic effect (Hedges’ g = −0.49, 95% CI: −0.73 to −0.25), with low-to-moderate heterogeneity (Q = 8.75, I^2^ = 42.9%, *p* = 0.119; Figure 2). In the pool-based program, longer exposure was associated with a greater absolute reduction in pain (ΔVAS = −2.52 at 24 weeks vs. −2.10 at 12 weeks), while standardized effects remained within the moderate range (d = −0.75 vs. −0.65).

#### 3.2.2. Resistance-Based Exercise

Across the nine resistance-based intervention arms (Table 2; RE, LBE), exercise intensity was categorized as low to moderate, based on Compendium-assigned MET values for resistance-type exercise ranging from 3.5 to 3.7 METs. Protocol duration ranged from 4 to 21 weeks, with sessions typically performed two to three times per week and lasting 30–60 min. Weekly exercise volume ranged from 280 to 666 MET·min/week (4.67 to 11.10 MET·h/week), and cumulative dose ranged from 1120 to 8880 MET·min (18.67 to 148.00 MET·h). All interventions showed reductions in VAS pain. Standardized within-arm pre–post effects ranged from d = −0.23 to d = −0.99, while absolute pain reduction ranged from −0.90 to −2.61. The pooled random-effects estimate showed a small-to-moderate analgesic effect (Hedges’ g = −0.48, 95% CI: −0.63 to −0.33), with low heterogeneity (Q = 8.53, I^2^ = 6.2%, *p* = 0.383; Figure 2). Overall, pain improvements were observed across the full dose range, without a clear monotonic increase in effect size with increasing cumulative MET exposure, suggesting that factors beyond cumulative workload may contribute to the analgesic response in resistance-based programs.

#### 3.2.3. Land-Based Physical Activity Programs

Exercise intensity ranged from moderate to vigorous across the four land-based physical activity program arms (Table 2; AE, LPA, HBE, FT), based on Compendium-assigned MET values ranging from 3.5 to 7.3 METs. Protocol duration ranged from 4 to 18 weeks, with weekly exercise volume ranging from 548 to 792 MET·min/week (9.13 to 13.20 MET·h/week) and cumulative dose ranging from 2736 to 14,256 MET·min (45.60 to 237.60 MET·h). All interventions demonstrated reductions in VAS pain, although the magnitude of improvement varied across protocols. Standardized within-arm pre–post effects ranged from d = −0.23 to d = −1.47, while absolute pain reduction ranged from −0.82 to −3.88. The pooled random-effects estimate indicated a moderate analgesic effect (Hedges’ g = −0.58, 95% CI: −0.98 to −0.18), although heterogeneity was substantial (Q = 9.76, I^2^ = 69.3%, *p* = 0.021; Figure 2). Overall, meaningful pain reductions were observed in both moderate- and higher-intensity programs, suggesting that land-based approaches may be effective across a broad MET-defined intensity spectrum. However, the variability in effect sizes and the substantial between-study heterogeneity indicate that factors such as program structure and supervision likely contribute to the observed analgesic response.

#### 3.2.4. Rehabilitation Interventions

Across the three rehabilitation intervention arms (Table 2; A-TeleRehab, S-TeleRehab, Rehab), exercise intensity was categorized as moderate based on the Compendium-assigned MET value of 4.3 METs. Protocol duration ranged from 4 to 10 weeks, with weekly exercise volume ranging from 516 to 774 MET·min/week (8.60 to 12.90 MET·h/week) and cumulative dose ranging from 3096 to 5160 MET·min (51.60 to 86.00 MET·h). All interventions demonstrated reductions in VAS pain. Standardized within-arm pre–post effects ranged from d = −0.26 to d = −1.37, while absolute pain reduction ranged from −0.85 to −2.50. The pooled random-effects estimate suggested the largest subgroup effect (Hedges’ g = −0.94, 95% CI: −1.63 to −0.25), although heterogeneity was considerable (Q = 13.58, I^2^ = 85.3%, *p* = 0.001; Figure 2). Taken together, these findings suggest that rehabilitation-based formats may be associated with clinically meaningful pain reduction, but the marked between-study variability indicates that differences in delivery mode, supervision, and program content likely influence outcomes in addition to cumulative workload.

#### 3.2.5. Complementary and Exercise-Adjunct Interventions

Across the eight complementary and exercise-adjunct intervention arms (Table 2; BALN, MIEP, MIEP+PNE, SPH), exercise intensity was categorized as very light to light, according to Compendium-assigned MET values ranging from 1.3 to 2.3 METs. Protocol duration ranged from 4 to 24 weeks, weekly exercise volume ranged from 104 to 184 MET·min/week (1.73 to 3.07 MET·h/week), and cumulative dose ranged from 416 to 3276 MET·min (6.93 to 54.60 MET·h). All interventions demonstrated reductions in VAS pain. Standardized within-arm pre–post effects ranged from d = −0.32 to d = −0.90, while absolute pain reduction ranged from −1.00 to −3.25. The pooled random-effects estimate indicated a moderate analgesic effect (Hedges’ g = −0.56, 95% CI: −0.72 to −0.39), with no observed heterogeneity (Q = 3.37, I^2^ = 0.0%, *p* = 0.849; Figure 2). Overall, these low-MET interventions were associated with relatively consistent pain improvements despite their low metabolic workload, suggesting that non-metabolic components, such as thermal exposure, relaxation training, graded movement, cognitive education, and imagery-based strategies, may also contribute meaningfully to the analgesic response.

### 3.3. Association Between Cumulative Exercise Dose and Pain Change in FM

**Figure 3:**
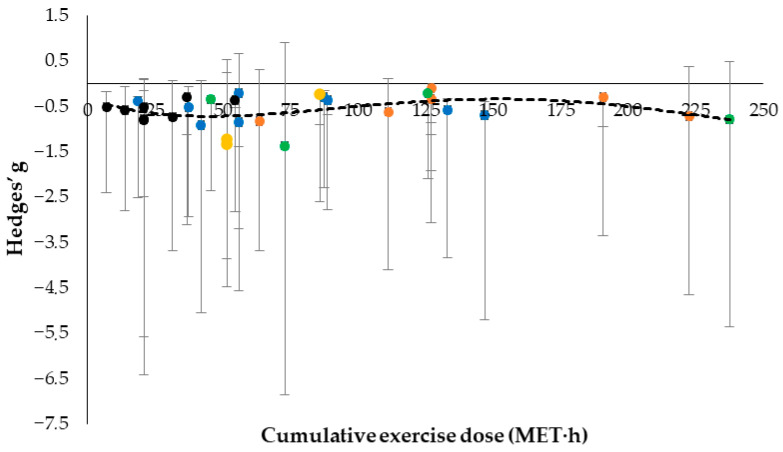
Association between cumulative exercise dose (MET·h) and analgesic effect size (Hedges’ g) across intervention arms (● Aquatic exercise; ● Resistance-based exercise; ● Land-based physical activity programs; ● Rehabilitation Interventions; ● Complementary and exercise-adjunct interventions). Negative values indicate pain reduction. Points are color-coded by exercise modality; error bars represent 95% confidence intervals. The dotted line shows an exploratory fourth-degree polynomial trendline (R^2^ = 0.1615). Because of the variability across exercise modalities and MET-based exposure, the plot illustrates general patterns rather than precise dose–response relationships.

To investigate whether the total amount of exercise prescribed explains the magnitude of the analgesic response in patients with FM, the cumulative dose was calculated as the total MET·h accrued over the intervention period. This was then plotted against the analgesic effect size (Hedges’ g) for each intervention arm (Figure 3). The scatter plot revealed substantial variability in analgesic responses across the entire dose range, with overlapping confidence intervals and similar effect sizes observed at both lower and higher cumulative doses. An exploratory fourth-degree polynomial trendline explained limited variance (R^2^ = 0.1615) and did not indicate a monotonic dose–response pattern. Consistent with this observation, an exploratory weighted linear analysis at the intervention-arm level showed that cumulative MET·h was not significantly associated with analgesic effect size (β = 0.0007, *p* = 0.507; R^2^ = 0.016). Taken together, these findings suggest that cumulative MET-based workload alone is a weak proxy for therapeutic response. These analyses should be interpreted as exploratory. Further insight into potential dose–response relationships may be provided by alternative modelling approaches (e.g., meta-regression).

### 3.4. Translation of Findings into a Clinical Tool

Due to variability in analgesic responses and an unclear monotonic dose–response relationship when using cumulative MET-based workload, we developed a preliminary symptom-responsive framework for exercise planning in fibromyalgia. This framework uses weekly pain feedback, expressed through VAS scores, together with short-term changes in pain and Talk Test thresholds, to support the adjustment of exercise intensity and volume on an individual basis. The concept is intended as a translational extension of the review findings, rather than a validated clinical tool.

### 3.5. Conceptual Structure of the Framework

In the proposed framework, pain intensity is assessed weekly using a 0–100 VAS. The change in pain intensity from one week to the next (ΔVAS), together with current symptom severity, is used to guide exercise progression. The framework uses the Talk Test as a practical method of limiting exercise intensity [29,30,31,32] and sets a weekly MET·min target responsive to symptoms within predefined safety boundaries. To enable comparison across activities, session planning is based on Compendium-derived MET values [12]. When pain is higher or worsening, the model applies a more conservative intensity cap and reduces the weekly exercise volume. When symptoms are stable or improving, gradual progression in weekly volume is permitted. The weekly target is distributed across aerobic, resistance and brief low-intensity sessions. The aim is to prioritize tolerability, pacing and adherence rather than uniform dose escalation. This framework was developed as a pragmatic, translational extension of the review findings and should be considered as generating hypotheses until it has been tested prospectively.

### 3.6. Preliminary Digital Implementation

To facilitate future clinical testing, this conceptual framework has been implemented as a preliminary web-based calculator integrated into a WordPress plugin developed by the USTEP Institute. The tool collects weekly VAS scores, calculates the change in VAS score (ΔVAS), applies an intensity cap based on the Talk Test and generates a weekly exercise plan responsive to symptoms, according to the conceptual rules described above. Currently, this calculator should be regarded as a prototype intended for future evaluation, rather than as an evidence-based clinical recommendation. Rigorous prospective studies are required to determine its feasibility, safety and effectiveness before it can be recommended for routine clinical use. The calculator is currently accessible via the following link [https://ustep.gr/vastt_vts_pro/ (21 March 2026)].

## 4. Discussion

This systematic review synthesized randomized controlled evidence on exercise-based interventions for fibromyalgia, examining whether a standardized, MET-based estimate of exercise dose could account for variation in pain outcomes. Across aquatic/pool-based, resistance-based, rehabilitation/telerehabilitation, land-based activity programs and complementary or exercise-adjunct interventions, exercise was consistently associated with reductions in pain. Pooled effects were typically in the small-to-moderate range, with larger improvements more commonly observed in structured or supervised formats. In most of the included trials, exercise was delivered alongside usual care rather than as monotherapy, so the observed effects probably reflect the additional benefit of exercise in routine management.

We evaluated whether cumulative intervention dose (cumulative MET·h) predicted the magnitude of analgesic response (Hedges’ g) by converting heterogeneous protocols into MET-defined workload metrics. The arm-level analyses showed substantial variability in analgesic effects across the full cumulative dose range, with overlapping confidence intervals and similar responses observed at both lower and higher doses (Figure 3). Exploratory weighted linear analysis did not reveal a significant association between cumulative MET·h and Hedges’ g (β = 0.0007, *p* = 0.507; R^2^ = 0.016). Similarly, the exploratory polynomial trendline explained only limited variance (R^2^ = 0.1615) and did not indicate a monotonic dose–response pattern. Together, these findings suggest that cumulative workload alone may be insufficient to explain pain outcomes in fibromyalgia. No consistent linear association was observed between a higher exercise dose and a greater analgesic benefit in the included intervention arms. Alternative explanations should also be considered, including non-linear dose response patterns and the influence of intervention structure or frequency.

In fibromyalgia, the lack of a clear dose–response gradient is biologically and clinically plausible, given that symptom sensitivity, impaired tolerance to physical load and fluctuating disease activity can constrain progression and reduce adherence. Large effects were not confined to a single dose range and were observed across a broad workload spectrum, supporting the idea that ‘more’ exercise is neither necessary nor sufficient for greater pain relief. Although moderate-to-vigorous programs can be highly beneficial for some individuals, particularly when structured and closely supervised, higher-intensity or higher-workload prescriptions may be intolerable for certain patients, especially those who are deconditioned or prone to symptom flare-ups. This highlights the importance of program characteristics (supervision, pacing strategies, cognitive/educational components, and exercise selection) and patient-level factors (baseline fitness, symptom stability, and adherence) in determining outcomes beyond cumulative workload.

In light of these findings, we developed a preliminary, clinically oriented, symptom-responsive exercise prescription framework that integrates weekly VAS, ΔVAS and Talk Test thresholds to adjust intensity and weekly volume within a safety envelope. This approach prioritizes tolerability and adherence while maintaining a standardized MET-based framework for planning external workload, offering a practical model for individualized exercise prescription in FM. These findings align with recent literature that supports the use of individualized, symptom-responsive exercise prescription approaches for populations with chronic pain and fibromyalgia [3,7,10]. Importantly, the proposed framework was not derived from a direct causal model, so it should be interpreted as an extension of the findings rather than as a validated intervention strategy.

### 4.1. Strengths and Limitations

This review has several strengths. It synthesizes RCTs spanning multiple exercise modalities and applies a consistent MET-based framework to standardize intensity and dose across heterogeneous protocols, enabling direct comparisons. The combined use of pooled meta-analytic estimates and arm-level exploration provides a pragmatic test of whether cumulative workload explains the analgesic response. Another strength is the translation of the findings into a framework that can be implemented to support clinical application.

Several limitations should be acknowledged. Firstly, MET values were estimated from exercise modality and intensity descriptors rather than being measured directly, which may have introduced imprecision in workload quantification. The substantial heterogeneity in program duration, supervision, structure, and co-interventions limits causal attribution to exercise dose and has likely contributed to variability between studies. In addition, the literature search was limited to PubMed, which is the largest and most authoritative database for medical research and indexes the vast majority of high-quality randomized controlled trials. While the use of a single database may have reduced the comprehensiveness of the study identification process, potentially resulting in the omission of relevant studies, the likelihood of missing eligible high-quality trials is considered low. Furthermore, the review protocol was not prospectively registered with PROSPERO. This reduces methodological transparency and increases the risk of selective reporting. The pooled effect estimates were based on within-group pre-post changes rather than direct between-group comparisons. This limits causal inference and the ability to attribute the observed changes exclusively to the exercise interventions. Therefore, the findings should be interpreted as reflecting overall changes in pain, rather than the comparative effectiveness of the interventions. Due to the modest number of subgroups and the heterogeneity of the interventions, sensitivity analyses and formal assessments of reporting bias (e.g., funnel plots) were not feasible, nor was grading the certainty of the evidence (e.g., GRADE). Dose response modelling should therefore be regarded as exploratory. Alternative analytical approaches, such as meta-regression models, could provide further insight into dose-response relationships and should be explored in future studies. Pain outcomes were predominantly assessed using VAS scores, and important potential confounders (e.g., medication use, psychological status, adherence, and baseline physical capacity) could not be consistently controlled for. Additionally, no sex-specific analyses were performed, meaning that potential sex-related differences in response to exercise could not be explored. Other clinically relevant variables, such as time since diagnosis, changes in medication during the intervention period and variability in the format of the intervention (e.g., supervised, home-based or telerehabilitation), were not consistently reported across studies. These variables could not be accounted for in the present synthesis and may have contributed to variability in pain outcomes. Therefore, residual confounding and publication bias cannot be excluded. Additionally, the symptom-responsive calculator described in this manuscript was not evaluated as part of the present review and should therefore be considered a preliminary translational concept rather than a validated clinical tool. Its clinical applicability, safety, and effectiveness require testing in rigorously designed prospective studies. Nevertheless, this synthesis provides a clinically relevant interpretation of exercise-induced analgesia in fibromyalgia and indicates that cumulative MET-based workload has limited explanatory value.

### 4.2. Clinical Implications and Digital Implementation

The negligible association between the cumulative MET-based dose and analgesic response suggests that increasing the dose alone may not be sufficient to optimize pain outcomes in fibromyalgia. Based on this, we explored a preliminary digital framework that converts weekly VAS and ΔVAS scores into a Talk Test-based intensity cap and a bounded weekly volume target. However, this framework has not yet been clinically validated and should be considered a hypothesis-generating tool, requiring prospective evaluation before it can be recommended for routine use.

## 5. Conclusions

Exercise interventions across multiple modalities were consistently associated with reductions in pain in individuals with FM, with pooled effects typically falling within the small-to-moderate range. Larger improvements were observed in structured or supervised programs. Analgesic benefits were observed across a wide dose range when exercise exposure was standardized using a MET-based dose. However, exploratory analyses showed limited explanatory value and no clear monotonic dose-response pattern. These findings suggest that therapeutic response may not be adequately explained by cumulative workload alone, and that factors such as supervision, pacing, adherence, symptom sensitivity, and intervention structure may also be influential. Given the exploratory nature of these analyses, a pain-responsive algorithm integrating weekly VAS, ΔVAS and Talk Test thresholds should be regarded as a preliminary framework for personalized exercise planning, pending prospective validation.

## Figures and Tables

**Table 1:** JBI randomized controlled trial critical appraisal checklist.

Author, Year	Q1	Q2	Q3	Q4	Q5	Q6	Q7	Q8	Q9	Q10	Q11	Q12	Q13	Score
Andrade et al. [14]														10
Assumpção et al. [15]														8
Bircan et al. [16]														10
Britto et al. [17]														13
Evcik et al. [18]														11
Fontaine et al. [19]														11
Giannotti et al. [20]														10
Kircali et al. [21]														11
Altan et al. [22]														9
Latorre Román et al. [23]														11
Rivas Neira et al. [24]														11
Silva et al. [25]														10
Timurtaş et al. [26]														10
Larsson et al. [27]														12
Häkkinen et al. [28]														9

Abbreviations: Green = yes, red = no, orange = unclear; Q1 = Clearly stated research question, Q2 = Appropriate study design, Q3 = Defined eligibility criteria and participant characteristics, Q4 = Adequate randomization procedure, Q5 = Blinding of participants or outcome assessors, Q6 = Reproducibility of the intervention description, Q7 = Completeness of follow-up and handling of attrition, Q8 = Appropriateness of statistical analysis, Q9 = Use of intention-to-treat analysis (if applicable), Q10 = Transparent reporting of results and uncertainty, Q11 = Consideration of confounding variables, Q12 = Declaration of funding sources or conflicts of interest, Q13 = Contribution to the field and contextual relevance.

**Table 2:** Exercise dose parameters and associated improvements in pain. The effect sizes reported are Cohen’s d, while the pooled estimates are Hedges’ g.

Authors	n	Age, Years	Exercise-Protocols	Duration		Frequency per Week	Weekly Dose (MET·h/Week)	VAS Score		95% Confidence Interval		Cohen’s d
				Weeks	Min/Session			Baseline	Follow-Up	Baseline	Follow-Up	
Bircan et al. [16]	13	48.3 ± 5.3	AE	8	25	3	9.13	6.07 ± 1.86	2.19 ± 1.88	4.9 to 7.19	1.05 to 3.33	−1.47
	13	46.0 ± 8.6	RE	8	30	3	5.25	5.21 ± 2.18	2.65 ± 1.41	3.98 to 6.53	1.80 to 3.50	−0.99
Britto et al. [17]	16	50.3 ± 6.1	WBE	8	60	3	15.90	7.84 ± 2.05	6.74 ± 2.28	6.75 to 8.93	5.53 to 7.95	−0.36
	17	46.2 ± 10.9	LBE	8	60	3	11.10	8.76 ± 1.41	7.72 ± 2.25	8.04 to 9.48	6.56 to 8.88	−0.39
Larsson et al. [27]	56	50.3 ± 9.7	RE	15	50	2	5.83	4.93 ± 2.39	3.86 ± 2.52	4.29 to 5.57	3.19 to 4.53	−0.31
Kircali et al. [21]	14	49.8 ± 9.1	MIEP+PNE	12	40	2	1.73	6.71 ± 1.94	3.86 ± 2.77	5.59 to 7.83	2.26 to 5.46	−0.84
	12	42.2 ± 10.9	MIEP	12	40	2	1.73	7.17 ± 1.99	3.92 ± 3.15	5.91 to 8.43	1.92 to 5.92	−0.87
Rivas Neira et al. [24]	18	48.0 ± 9.0	AqE	12	60	3	15.90	4.73 ± 1.78	3.53 ± 3.26	3.84 to 5.56	1.91 to 5.15	−0.32
	17	52.0 ± 9.0	LBE	12	60	3	11.10	5.73 ± 2.07	3.97 ± 2.07	4.67 to 6.97	2.91 to 5.03	−0.60
Assumpção et al. [15]	14	47.9 ± 5.3	STE	12	40	2	3.07	5.60 ± 1.80	4.60 ± 2.60	4.56 to 6.64	3.10 to 6.10	−0.32
	15	45.7 ± 7.7	RE	12	40	2	4.67	5.30 ± 2.50	4.40 ± 3.00	3.97 to 6.63	2.80 to 6.00	−0.23
Andrade et al. [14]	25	48.0 ± 8.0	AqE	16	45	2	7.95	5.80 ± 2.70	5.40 ± 2.40	4.73 to 6.87	4.45 to 6.35	−0.11
Fontaine et al. [19]	46	46.4 ± 11.6	LPA	12	60	3	10.50	5.46 ± 2.56	4.64 ± 2.42	4.70 to 6.22	3.92 to 5.36	−0.23
Giannotti et al. [20]	20	52.8 ± 10.6	Rehab	10	60	2	8.60	6.10 ± 2.07	5.25 ± 2.47	5.13 to 7.07	4.09 to 6.41	−0.26
Häkkinen et al. [28]	11	39.0 ± 6.0	RE	21	60	2	7.00	4.80 ± 2.50	2.40 ± 1.90	3.12 to 6.48	1.12 to 3.68	−0.76
Timurtaş et al. [26]	33	47.0 ± 13.7	A-TeleRehab	4	60	3	12.90	6.81 ± 1.17	4.31 ± 1.40	6.40 to 7.22	3.81 to 4.81	−1.37
	33	46.2 ± 14.3	S-TeleRehab	4	60	3	12.90	6.72 ± 1.28	4.66 ± 1.00	6.27 to 7.17	4.31 to 5.01	−1.27
Evcik et al. [18]	31	43.8 ± 7.7	AqE	4	60	3	15.90	6.20 ± 1.70	4.20 ± 1.60	5.58 to 6.82	3.61 to 4.79	−0.86
	30	42.8 ± 7.6	HBE	4	60	3	11.40	6.10 ± 1.90	5.10 ± 1.90	5.39 to 6.81	4.39 to 5.81	−0.37
Altan et al. [22]	24	43.1 ± 6.4	PBE	12	35	3	9.28	7.91 ± 1.81	5.81 ± 2.70	7.15 to 8.67	4.67 to 6.95	−0.65
	22	43.9 ± 6.3	BALN	12	35	3	2.63	7.50 ± 1.82	5.63 ± 1.62	6.96 to 8.31	4.91 to 6.35	−0.77
	24	43.1 ± 6.4	PBE	24	35	3	9.28	7.91 ± 1.81	5.39 ± 2.84	7.15 to 8.67	4.19 to 6.59	−0.75
	22	43.9 ± 6.3	BALN	24	35	3	2.28	7.50 ± 1.82	6.36 ± 2.33	6.96 to 8.31	5.33 to 7.39	−0.39
Silva et al. [25]	30	44.9 ± 10.3	RE	4	40	2	4.67	6.67 ± 1.47	5.56 ± 2.32	6.12 to 7.22	4.69 to 6.43	−0.40
	30	49.4 ± 8.3	SPH	4	40	2	1.73	6.27 ± 1.46	5.16 ± 1.51	5.72 to 6.82	4.60 to 5.72	−0.53
	30	44.9 ± 10.3	RE	8	40	2	4.67	6.67 ± 1.47	5.23 ± 2.16	6.12 to 7.22	4.42 to 6.04	−0.55
	30	49.4 ± 8.3	SPH	8	40	2	1.73	6.27 ± 1.46	4.90 ± 1.73	5.72 to 6.82	4.25 to 5.55	−0.61
	30	44.9 ± 10.3	RE	12	40	2	4.67	6.67 ± 1.47	4.06 ± 2.58	6.12 to 7.22	3.10 to 5.02	−0.88
	30	49.4 ± 8.3	SPH	12	40	2	1.73	6.27 ± 1.46	5.10 ± 1.62	5.72 to 6.82	4.50 to 5.70	−0.54
Latorre Román et al. [23]	20	51.7 ± 9.5	FT	18	60	3	13.20	9.40 ± 1.66	6.47 ± 3.10	8.62 to 10.18	5.02 to 7.92	−0.83

Abbreviations: AE = aerobic exercise; AqE = aquatic exercise; A-TeleRehab = asynchronous telerehabilitation; BALN = balneotherapy; FT = functional training; HBE = home-based exercise; LBE = land-based exercise; LPA = lifestyle physical activity; MIEP = motor imagery-based exercise; MIEP+PNE = motor imagery-based exercise protocol and pain neuroscience education; PBE = pool-based exercise; RE = resistance exercise; Rehab = rehabilitation; SPH = sophrology; S-TeleRehab = synchronous telerehabilitation; WBE = water-based exercise.

## Data Availability

No new data were created or analyzed in this study.

## Supplementary Materials

The following supporting information can be downloaded at: https://www.mdpi.com/article/10.3390/brainsci16040365/s1, Table S1: PRISMA checklist.

## Abbreviations

The following abbreviations are used in this manuscript:

A	Aerobic sessions
AE	Aerobic exercise
AqE	Aquatic exercise
A-TeleRehab	Asynchronous telerehabilitation
BALN	Balneotherapy
CI	Confidence interval
FM	Fibromyalgia
FT	Functional training
g	Hedges’ g
HBE	Home-based exercise
I^2^	I-squared statistic
JBI	Joanna Briggs Institute
LBE	Land-based exercise
LPA	Lifestyle physical activity
M	Mini sessions
MET·h	MET-hours
MET·min	MET-minutes
METs	Metabolic equivalents
MIEP	Motor imagery-based exercise protocol
PBE	Pool-based exercise
PNE	Pain neuroscience education
PRISMA	Preferred Reporting Items for Systematic Reviews and Meta-Analyses
Q	Cochran’s Q
R	Resistance sessions
RCT	Randomized controlled trial
RE	Resistance exercise
Rehab	Rehabilitation
SD	Standard deviation
SMD	Standardized mean difference
SPH	Sophrology
S-TeleRehab	Synchronous telerehabilitation
TT	Talk Test
VAS	Visual Analogue Scale
WBE	Water-based exercise

## References

[1] Clauw D.J. (2014). Fibromyalgia: A Clinical Review. JAMA.

[2] Häuser W., Ablin J., Fitzcharles M.A., Littlejohn G., Luciano J.V., Usui C., Walitt B. (2015). Fibromyalgia. Nat. Rev. Dis. Primers.

[3] Macfarlane G.J., Kronisch C., Dean L.E., Atzeni F., Häuser W., Fluß E., Choy E., Kosek E., Amris K., Branco J. (2017). EULAR revised recommendations for the management of fibromyalgia. Ann. Rheum. Dis..

[4] O’Brien A.T., Deitos A., Triñanes Pego Y., Fregni F., Carrillo-de-la-Peña M.T. (2018). Defective Endogenous Pain Modulation in Fibromyalgia: A Meta-Analysis of Temporal Summation and Conditioned Pain Modulation Paradigms. J. Pain.

[5] Pacheco-Barrios K., Filardi R.M., González-González L.F., Park N., Petrus F.Q., Navarro-Flores A., Di-Bonaventura S., Alves L.G., Queiroz F., Fregni F. (2024). The Link between Endogenous Pain Modulation Changes and Clinical Improvement in Fibromyalgia Syndrome: A Meta-Regression Analysis. Biomedicines.

[6] Bidonde J., Busch A.J., Schachter C.L., Overend T.J., Kim S.Y., Góes S.M., Boden C., Foulds H.J. (2017). Aerobic exercise training for adults with fibromyalgia. Cochrane Database Syst. Rev..

[7] Busch A.J., Webber S.C., Richards R.S., Bidonde J., Schachter C.L., Schafer L.A., Danyliw A., Sawant A., Dal Bello-Haas V., Rader T. (2013). Resistance exercise training for fibromyalgia. Cochrane Database Syst. Rev..

[8] Kim S.Y., Busch A.J., Overend T.J., Schachter C.L., van der Spuy I., Boden C., Góes S.M., Foulds H.J., Bidonde J. (2019). Flexibility exercise training for adults with fibromyalgia. Cochrane Database Syst. Rev..

[9] Wang C., Schmid C.H., Fielding R.A., Harvey W.F., Reid K.F., Price L.L., Driban J.B., Kalish R., Rones R., McAlindon T. (2018). Effect of tai chi versus aerobic exercise for fibromyalgia: Comparative effectiveness randomized controlled trial. BMJ.

[10] Sosa-Reina M.D., Nunez-Nagy S., Gallego-Izquierdo T., Pecos-Martín D., Monserrat J., Álvarez-Mon M. (2017). Effectiveness of Therapeutic Exercise in Fibromyalgia Syndrome: A Systematic Review and Meta-Analysis of Randomized Clinical Trials. Biomed Res Int..

[11] Häuser W., Brähler E., Ablin J., Wolfe F. (2021). Modified 2016 American College of Rheumatology Fibromyalgia Criteria, the Analgesic, Anesthetic, and Addiction Clinical Trial Translations Innovations Opportunities and Networks-American Pain Society Pain Taxonomy, and the Prevalence of Fibromyalgia. Arthritis Care Res..

[12] Ainsworth B.E., Haskell W.L., Herrmann S.D., Meckes N., Bassett D.R., Tudor-Locke C., Greer J.L., Vezina J., Whitt-Glover M.C., Leon A.S. (2011). 2011 Compendium of Physical Activities: A second update of codes and MET values. Med. Sci. Sports Exerc..

[13] Aromataris E., Munn Z. (2020). JBI Manual for Evidence Synthesis. JBI. https://synthesismanual.jbi.global.

[14] Andrade C.P., Zamunér A.R., Forti M., Tamburús N.Y., Silva E. (2019). Effects of aquatic training and detraining on women with fibromyalgia: Controlled randomized clinical trial. Eur. J. Phys. Rehabil. Med..

[15] Assumpção A., Matsutani L.A., Yuan S.L., Santo A.S., Sauer J., Mango P., Marques A.P. (2018). Muscle stretching exercises and resistance training in fibromyalgia: Which is better? A three-arm randomized controlled trial. Eur. J. Phys. Rehabil. Med..

[16] Bircan C., Karasel S.A., Akgün B., El O., Alper S. (2008). Effects of muscle strengthening versus aerobic exercise program in fibromyalgia. Rheumatol. Int..

[17] Britto A., Rodrigues V., Dos Santos A.M., Rizzini M., Britto P., Britto L., Garcia J.B.S. (2020). Effects of water- and land-based exercises on quality of life and physical aspects in women with fibromyalgia: A randomized clinical trial. Musculoskelet. Care.

[18] Evcik D., Yigit I., Pusak H., Kavuncu V. (2008). Effectiveness of aquatic therapy in the treatment of fibromyalgia syndrome: A randomized controlled open study. Rheumatol. Int..

[19] Fontaine K.R., Conn L., Clauw D.J. (2010). Effects of lifestyle physical activity on perceived symptoms and physical function in adults with fibromyalgia: Results of a randomized trial. Arthritis Res. Ther..

[20] Giannotti E., Koutsikos K., Pigatto M., Rampudda M.E., Doria A., Masiero S. (2014). Medium-/long-term effects of a specific exercise protocol combined with patient education on spine mobility, chronic fatigue, pain, aerobic fitness and level of disability in fibromyalgia. BioMed Res. Int..

[21] Kircali S., Özcan Ö.Ö., Karahan M. (2024). Pain neuroscience education and motor imagery-based exercise protocol for patients with fibromyalgia: A randomized controlled trial. Brain Behav..

[22] Altan L., Bingöl U., Aykaç M., Koç Z., Yurtkuran M. (2004). Investigation of the effects of pool-based exercise on fibromyalgia syndrome. Rheumatol. Int..

[23] Román P.Á.L., Santos E., Campos M.A., García-Pinillos F. (2015). Effects of functional training on pain, leg strength, and balance in women with fibromyalgia. Mod. Rheumatol..

[24] Neira S.R., Marques A.P., Cervantes R.F., Pillado M.T.S., Costa J.V. (2024). Efficacy of aquatic vs land-based therapy for pain management in women with fibromyalgia: A randomised controlled trial. Physiotherapy.

[25] Silva H.J.A., Júnior J.C.A., de Oliveira F.S., Oliveira J.M.P., Dantas G.A.F., Lins C.A.A., de Souza M.C. (2019). Sophrology versus resistance training for treatment of women with fibromyalgia: A randomized controlled trial. J. Bodyw. Mov. Ther..

[26] Timurtaş E., Hüzmeli İ., Demirbüken İ., Polat M.G. (2025). Clinical outcomes of asynchronous telerehabilitation through a mobile app are equivalent to synchronous telerehabilitation in patients with fibromyalgia: A randomized control study. BMC Musculoskelet. Disord..

[27] Larsson A., Palstam A., Löfgren M., Ernberg M., Bjersing J., Bileviciute-Ljungar I., Gerdle B., Kosek E., Mannerkorpi K. (2015). Resistance exercise improves muscle strength, health status and pain intensity in fibromyalgia—A randomized controlled trial. Arthritis Res. Ther..

[28] Häkkinen A., Häkkinen K., Hannonen P., Alen M. (2001). Strength training induced adaptations in neuromuscular function of premenopausal women with fibromyalgia: Comparison with healthy women. Ann. Rheum. Dis..

[29] Persinger R., Foster C., Gibson M., Fater D.C., Porcari J.P. (2004). Consistency of the talk test for exercise prescription. Med. Sci. Sports Exerc..

[30] Reed J.L., Pipe A.L. (2014). The talk test: A useful tool for prescribing and monitoring exercise intensity. Curr. Opin. Cardiol..

[31] Thompson P.D., Arena R., Riebe D., Pescatello L.S., American College of Sports Medicine (2013). ACSM’s new preparticipation health screening recommendations from ACSM’s guidelines for exercise testing and prescription, ninth edition. Curr. Sports Med. Rep..

[32] Quinn T.J., Coons B.A. (2011). The Talk Test and its relationship with the ventilatory and lactate thresholds. J. Sports Sci..

